# Imaging neuron-glia interactions in the enteric nervous system

**DOI:** 10.3389/fncel.2013.00183

**Published:** 2013-10-21

**Authors:** Werend Boesmans, Michiel A. Martens, Nathalie Weltens, Marlene M. Hao, Jan Tack, Carla Cirillo, Pieter Vanden Berghe

**Affiliations:** ^1^Laboratory for Enteric NeuroScience (LENS), Translational Research Center for GastroIntestinal Disorders, University of Leuven, Leuven, Belgium; ^2^Translational Research Center for GastroIntestinal Disorders (TARGID), Department of Clinical and Experimental Medicine, University of Leuven, Leuven, Belgium

**Keywords:** enteric neuron, enteric glia, calcium, synaptic, GCaMP

## Abstract

The enteric nervous system (ENS) is a network of neurons and glia within the wall of the gastrointestinal tract that is able to control many aspects of digestive function independently from the central nervous system. Enteric glial cells share several features with astrocytes and are closely associated with enteric neurons and their processes both within enteric ganglia, and along interconnecting fiber bundles. Similar to other parts of the nervous system, there is communication between enteric neurons and glia; enteric glial cells can detect neuronal activity and have the machinery to intermediate neurotransmission. However, due to the close contact between these two cell types and the particular characteristics of the gut wall, the recording of enteric glial cell activity in live imaging experiments, especially in the context of their interaction with neurons, is not straightforward. Most studies have used calcium imaging approaches to examine enteric glial cell activity but in many cases, it is difficult to distinguish whether observed transients arise from glial cells, or neuronal processes or varicosities in their vicinity. In this technical report, we describe a number of approaches to unravel the complex neuron-glia crosstalk in the ENS, focusing on the challenges and possibilities of live microscopic imaging in both animal models and human tissue samples.

## Introduction

Many aspects of gastrointestinal function are controlled without major inputs from the brain. Instead, the enteric nervous system (ENS), a ganglionated neuronal network that resides within the gut wall, autonomously controls gastrointestinal motility, secretion, and blood flow (Furness, 2000). The ENS develops from neural crest cells that migrate and proliferate extensively to eventually form a network of interconnected ganglia throughout the entire length of the gut (Sasselli et al., 2012; Obermayr et al., 2013). Similar to other parts of the nervous system, the ENS comprises both a neuronal and a glial component. Enteric glial cells are located in close contact with enteric neurons within the ganglia, along interganglionic connectives of the myenteric and submucosal plexus, and can also be found in the extraganglionic layers of the gut wall (Gershon and Rothman, 1991). In contrast to other parts of the peripheral nervous system, the ENS is quite exceptional: it lacks coats of connective tissue that surround nerve cell bodies and fibers and is therefore more reminiscent to the central nervous system (Cook and Burnstock, 1976; Gabella, 1981). Enteric glial cells share many phenotypical features with astrocytes, and were for a long time also believed to function mainly as support cells for neurons. However, in the last two decades this dogma has gradually been abandoned and considerable progress has been made in understanding enteric glial function, most of which has been covered in recent reviews (De Giorgio et al., 2012; Gulbransen and Sharkey, 2012; Neunlist et al., 2013).

Enteric glial cells are considered to be active partners in ENS function (Ruhl et al., 2004). They display dynamic responses to neuronal inputs and have the apparatus to sequester and release neuro-active factors. Nonetheless, whether enteric glial cells indeed regulate synaptic transmission in a physiological context such as during gastrointestinal motility patterns is not known. Furthermore, before the concept of the “tripartite synapse”—with the glial cell as a full synaptic partner (Perea et al., 2009)—can also be established in the ENS, it needs to be elucidated what “gliotransmitters” are released by enteric glia in response to neuronal activity, and perhaps act on neighboring pre- and postsynaptic elements. Also a better understanding of the initial steps in such reciprocal neuron-glia communication, i.e., the integration of neuronal inputs to enteric glia, is vital. However, the detection of enteric glial activity, especially when their interaction with enteric neurons is targeted, is not unambiguous. The close proximity between enteric glia and neurons and their processes makes optical discrimination between signals originating from specific cells particularly challenging.

In this technical report, we describe a number of approaches to disentangle the complex neuron-glia crosstalk in the ENS, in both animal models and human tissue. We discuss the techniques that have been used to examine neuron-glia interactions in the animal and human ENS with an emphasis on live intracellular calcium concentration ([Ca^2+^]_i_) imaging and present some novel analysis tools that serve this purpose.

## Neurons and glia: close neighbors in the gut

**Figure 1:**
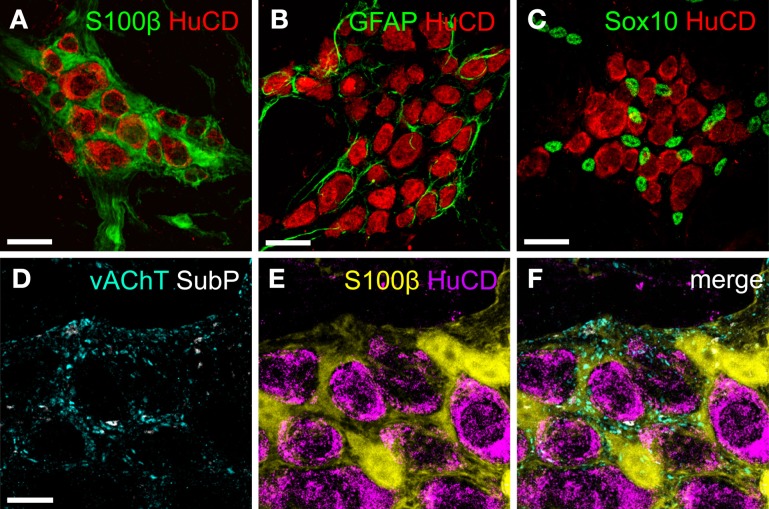
**The relation between enteric neurons and glia visualized by immunohistochemistry. (A,C)** Maximum projection of colonic myenteric ganglia of mice stained for the pan-neuronal marker HuCD (red) and enteric glial cell markers (green) S100β **(A)**, GFAP **(B)**, and Sox10 **(C)**. **(D,E)** Maximum projection composed of a stack of two confocal images of a mouse colonic myenteric ganglion labeled with antibodies for S100β (glia, yellow) and HuCD (neurons, magenta) and for vAChT (cyan) and substance P (grays) revealing the close apposition of neuronal fibers and varicosities with enteric glial cells **(F)**. Scale bars: 25μm **(A–C)**, 10μm **(D)**.

Enteric glial cells resemble astrocytes in several ways, including the expression of the intermediate filament glial fibrillary acidic protein (GFAP) (Jessen and Mirsky, 1980) and the Ca^2+^ binding protein, S100β (Ferri et al., 1982). The intimate association between glia and neurons within enteric ganglia has been revealed by co-immunolabeling of gut tissue with GFAP or S100β with neuronal markers, such as HuC/D (Figures 1A,B). In addition, the transcription factor SRY box–containing gene 10 (Sox10), which is expressed by multipotent ENS precursors (Paratore et al., 2002; Bondurand et al., 2003), is also expressed by mature enteric glia (Young et al., 2003), and is ideally suited for quantification purposes because of its selective nuclear localization (Figure 1C) (Hoff et al., 2008). Enteric glial cells closely embrace nerve fibers and varicose release sites both within enteric ganglia and along the interconnective fiber tracts (Figures 1D–F) (Hanani and Reichenbach, 1994; Vanden Berghe and Klingauf, 2007). As a consequence, the optical segregation of signals arising from either neurons or glia in live imaging experiments is not straightforward.

Microscopic imaging techniques have provided invaluable information about several aspects of neuronal signaling in both the developing and adult ENS (Schemann et al., 2002; Vanden Berghe et al., 2008; Hao et al., 2012). In particular, chemo- and mechanosensitivity of various classes of enteric neurons has been uncovered using both voltage-sensitive and Ca^2+^ indicator dyes (Smith et al., 2007; Schemann and Mazzuoli, 2010). Strong evidence for communication between enteric neurons and glia comes from a series of studies using live imaging in both *ex vivo* wholemount preparations of gut and in cell cultures of the ENS (Gomes et al., 2009; Gulbransen and Sharkey, 2009; Gulbransen et al., 2010). In addition, in a study by Broadhead et al., interaction between neuronal and glial cells was shown following spontaneous or induced physiological activity (Broadhead et al., 2012). In all studies, purinergic signaling pathways have been identified as the primary mechanism of transmission.

## Live imaging of neuron-glia interactions: *in vitro* vs. *ex vivo*

**Figure 2:**
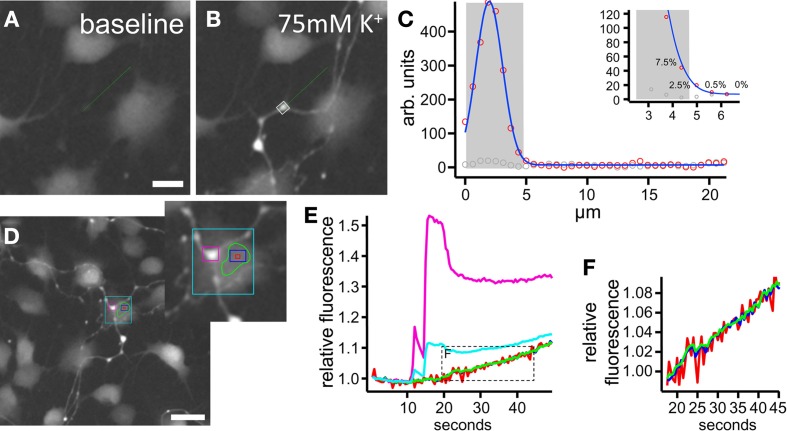
**Quantification of Fluo4 signals in regions of interest (ROI). (A**–**C**) Example of a neuronal fiber and bouton at rest **(A)** and depolarized with 75 mM K^+^ **(B)**. In **(C)** the amplitude (at rest in gray, depolarized in red circles) along the line marked green in **(A)** and **(B)** is plotted. As seen, especially in the inset, the signal drops sharply outside the intuitively drawn ROI [gray shade bar in **(C)** and gray box in **(B)**]: the last pixel included contains 7.5% of the signal's maximal amplitude, while the first pixel outside the ROI only contains 2.5%. **(D–F)** Analysis of a situation in which a neuronal fiber crosses a glial cell. The cyan region of interest includes a mixture of both neuronal (purple) and glial (red, blue, green) information. Smaller regions of interest (rectangular or polygon shaped) away from the nerve fiber identify pure glial signals with improving signal to noise ratios **(F)** for larger areas included. Scale bars: 10μm **(A,B)**, 20μm **(D)**.

Optical information generated by multiple sources is always convoluted due to the diffraction limitations of optical microscopy. To address this, we estimated the contribution of any given signal in the pixels directly neighboring a structure of interest as recorded by a widefield fluorescence microscope equipped with a CCD camera. Primary ENS cultures (including both neurons and glia) were loaded with the Ca^2+^ indicator dye, Fluo4-AM, and stimulated by 75 mM K^+^ depolarization (Figure 2). To measure the change in fluorescence, a region of interest (ROI) was drawn over a neuronal bouton. We found that even with limited optical resolution (widefield, 20×, NA = 0.75, pixel width: 623 nm), the signal contribution drops sharply outside of any structure that can be picked out intuitively by an observer (Figure 2C). Simple rectangular ROIs are sufficient to calculate the cellular signals and polygon-shaped ROIs do improve signal to noise ratios due to inclusion of larger cell areas (Figures 2D–F). Thus, at least in *in vitro* experiments, careful drawing of ROIs at least 1μm away from each other may be sufficient to separate signals coming from structures situated in each other's vicinity.

The ENS, due to its planar organization in *ex vivo* preparations, is very attractive to investigate with imaging techniques. However, the assumption of 2D structure is only valid when entire cells are considered. Once synaptic contacts and cellular processes are of interest, the analysis faces all the technical problems that are associated with 3D organization and ROIs will easily incorporate scattered light emanating from structures close by. Hence, the intimate relationship between enteric glia, neurons and their processes (Figure 1) entails the risk that signals arising from enteric varicosities and fibers are interpreted as being of glial origin. This is an important confounding factor that may cause the false impression that glial cells respond as fast to electrical and depolarizing stimuli as do neurons. Therefore, to make use of the fundamental physiological difference between neurons and electrically passive non-excitable glia (Hanani et al., 2000; Gulbransen and Sharkey, 2012) we suggest, in combination with using lenses with sharp focal depths, to consistently apply known stimuli (e.g., electrical stimulation) to identify neuronal structures, which then serve as a guide to draw regions at least 1μm away from other structures in order to minimize false interpretation.

The clear delineation of the cells and compartments of interest in *ex vivo* gut preparations is further complicated by the fact that these ganglia are on a contractile muscle layer, which even with pharmacological inhibition and mechanical restraining can still cause movement artifacts. This further complicates accurate analysis, especially of smaller structures and cell compartments (e.g., varicosities, parts of glial processes). To correct for residual movements, we use translation stabilization routines (Bisschops et al., 2006; Gallego et al., 2008), which recently have been expanded to also correct for more complex movements like rotation and torque.

## Differentiating between neuronal and glial responses: response timing and shape

Although originally not intended for studying neuron-glia interactions, data from pioneering studies showing that several neuroligands can elicit Ca^2+^ transients in cultured enteric glial cells have already indicated the potential for glial participation in enteric neurotransmission (Kimball and Mulholland, 1996; Zhang et al., 1997, 1998; Garrido et al., 2002). In an alternative approach to directly measure the sensitivity of enteric glial cells to neuronal transmitters, we used an immortalized rat enteric glia cell line (CRL-2690) (Ruhl et al., 2001) and confirmed that neurotransmitters known to elicit fast excitatory potentials in enteric neurons can directly induce [Ca^2+^]_i_ changes in enteric glial cells (Boesmans et al., 2013). In contrast, enteric glia did not respond to high K^+^ depolarization. The absence of neurons in these cultures obviously eliminates the problems illustrated above, but also excludes the possibility that these cells are activated secondary to neuronal activation. However, in mixed cultures of neurons and glia this is not the case (Gomes et al., 2009). By specifically stimulating enteric neurons while monitoring the secondary glial responses, an adenosine tryphosphate (ATP)-dependent paracrine communication pathway between enteric neurons and glia was revealed.

**Figure 3:**
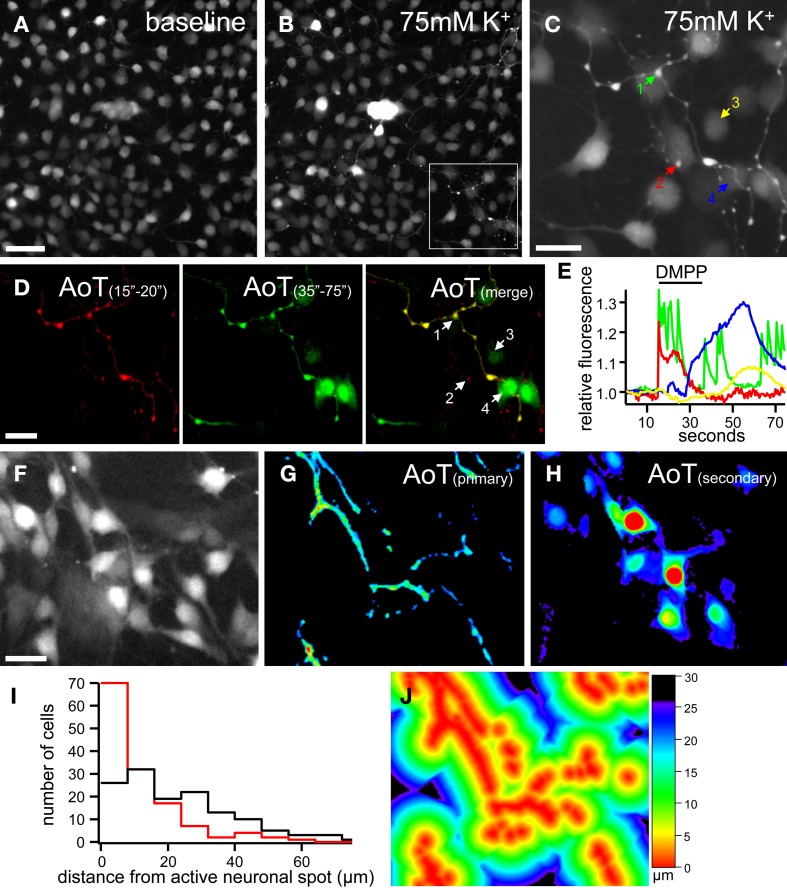
**Ca^2+^ imaging of enteric neuron-glia interactions in primary enteric nervous system cultures. (A,B)** Gray scale images of a patch of cultured mouse enteric neurons and glia loaded with Fluo4 at rest **(A)** and depolarized by 75 mM K^+^ application 5 s, **(B)**. Note the large increase in fluorescence of the group of neuronal cell bodies in the center of the image. **(C)** Magnification of the frame indicated in **(B)** showing in detail the neuronal fibers and varicosities as depolarized by 75 mM K^+^. **(D)** Activity over time (AoT) images of the primary (red) and secondary (green) responses to nicotinic receptor stimulation (DMPP, 10μM, 20 s) of the same group of cells as indicated in **(C)**. **(E)** Ca^2+^ responses of 2 neuronal boutons (1, green and 2, red) and 2 glial cells (3, yellow and 4, blue) upon DMPP application [color-coded numbers in **(C)** and numbers in **(D)**]. Note the fast upstroke and reverberating activity in neuronal varicosities and the delayed secondary Ca^2+^ transients in enteric glial cells. See also Supplementary movie 1. **(F)** Gray scale image of a patch of cultured mouse enteric neurons and glia loaded with Fluo4 at rest. **(G,H)** AoT images of the same patch of cells as in **(F)** in which neuronal fibers responding directly **(G)** and cells displaying a slow Ca^2+^ response **(H)** to electrical field stimulation (2 s, 20 Hz) are shown. **(I)** Histogram displaying the distances (μm) from an active neuronal component to cells with (red, *n* = 135) and without (black, *n* = 134) secondary responses to nerve stimulation (*p* < 0.05, Fisher's exact). **(J)** Image of the same cells as in **(F)** in which the distance from each pixel to an active neuronal component is color-coded. Scale bars: 50μm **(A,F)**, 20μm **(C,D)**.

This typical ATP sensitivity was also found in enteric neuron-glia co-cultures obtained from adult mouse gut where the neuronal and glial Ca^2+^ fingerprint was used to identify specific cell types (Laranjeira et al., 2011). Indeed, enteric neurons display a strong and fast Ca^2+^ response to high K^+^ depolarization, electrical field stimulation (EFS) and the nicotinic agonist dimethylphenylpiperazinium (DMPP). Enteric glial cells, on the other hand, do not respond to these stimuli directly, but show delayed responses that can be modulated by intervening with several components of purinergic signaling (Gomes et al., 2009; Laranjeira et al., 2011) (Figure 3). Due to these timing differences, it is possible to construct “activity-over-time” (AoT, Figure 3D) images that identify cells which exhibit a change in fluorescence intensity above baseline noise. These images appear similar to immunostainings, but a physiological response is represented instead of the structural information (custom developed algorithm in Igor Pro, Wavematrics).

To further characterize the timing of the responses, we developed a routine in which the shortest distance to an active neuronal fiber was computed and transformed in a color coded image (Figures 3F–J). The computation of distance can be performed either on an immunochemical staining or on one of the AoT images generated from live recordings. In this way we are able to test whether secondary responses, for instance responses in glial cells, emerge earlier if they are physically closer to active neuronal fibers. We found that the timing of glial responses does correlate with spatial aspects since cells closer to a neuronal component have a higher likelihood of responding to a neuronal stimulus (Figure 3I), thus further corroborating the fact that it is a diffusible factor that mediates the communication from enteric neurons to glia.

**Figure 4:**
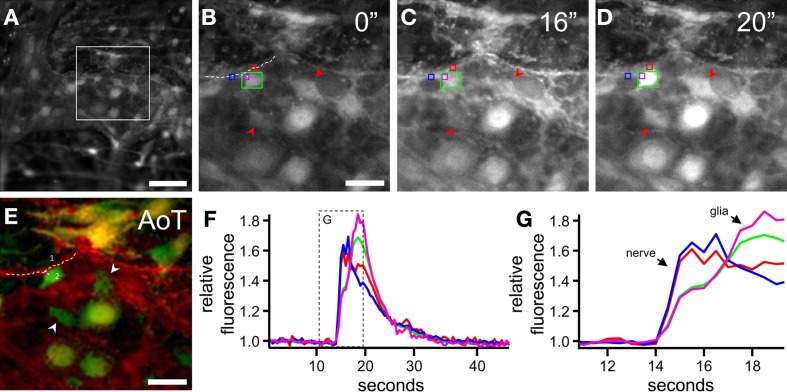
**Detection of neuronal vs. glial signals in *ex vivo* enteric nervous system preparations. (A)** Gray scale image of a colonic myenteric plexus ganglion loaded with Fluo4 at rest. **(B–D)** Magnification of the square region marked in **(A)** before **(B)** and after **(C,D)** electrical stimulation (ETS, 2 s, 20 Hz) of an interganglionic connective. **(E)** Activity over time (AoT) image of the region in which pixels responding immediately (red) or with a delay (green) to ETS are false colored. Arrowheads point to enteric glial cells that display a Ca^2+^ transient secondary to neuronal stimulation. Dashed line (1) and (2) mark the neuronal fiber and glia cell used in **(F)** and **(G)**. (**F**) Fluo4 traces of the regions of interest (color-coded in **B**–**D**) showing responses upon ETS. **(G)** Magnification of the squared box in **(F)**. Although the initial increase in the purple and green trace is due to a neuronal fiber (1) crossing the glial cell (2) it is still possible, because of the differences in upstroke speed to distinguish between neuronal and glial cell types. Scale bars: 50μm **(A)**, 20μm **(B,E)**.

Taking all these technical issues into consideration, it is possible to isolate the net responses from neurons and glial cells in complex tissues. This reveals that the Ca^2+^ transients in both cell types have typical shapes and kinetics to stimuli like EFS, high K^+^ depolarization and fast neurotransmitters: in neurons, fast and linear upstrokes reaching their maximum in a couple of seconds are followed by a (bi)-exponential decay, while in glia a secondary close-to-Gaussian shaped response is observed. This typical fingerprint can thus be used also in tissue to identify different cell types (Figure 4). Provided that sufficient spatial resolution is achieved, fast imaging approaches (Michel et al., 2011; Martens and Vanden Berghe, 2012) can help discriminating between neuronal and glial signals because the extra data points allow more reliable fitting of the response upstroke.

It is of note that enteric glial cells display higher baseline fluorescence after Fluo4 loading compared to neurons (arbitrary fluorescence units, neurons: 260.7 vs. glia: 339.6, *p* < 0.05, *n* = 103, data from three animals, unpaired *t*-test), a difference that is more pronounced in *ex-vivo* tissue preparations in comparison to primary culture. Using the ratiometric dye Fura2, we tested whether this difference was due to higher resting level of intracellular Ca^2+^. We found the differences (340/380 ratio, neurons: 0.3442 vs. glia: 0.3623; *p* < 0.05, *n* = 120, data from three animals, unpaired *t*-test) to be only very small and definitely not sufficient to explain the large differences in resting Fluo4 fluorescence. This was further confirmed by using Rhod2, a non-ratiometric Ca^2+^ indicator with an even higher K_d_, which is classically used in astrocyte research (Mulligan and Macvicar, 2004; Takano et al., 2006) and can also be used to load enteric glial cells (Gulbransen and Sharkey, 2009). Again, higher resting levels were observed in glial cells compared to neurons (arbitrary fluorescence units, neurons: 330.8 vs. glia: 419.4, *p* < 0.05, *n* = 113, data from three animals, unpaired *t*-test). Taken together, this suggests that glial cells take up and/or metabolize the AM ester more easily, which is probably a reflection of a higher metabolism.

## Novel genetic tools for studying enteric neuron-glia interactions

Apart from identification during live recordings and *post-hoc* analysis, many new genetic tools are available to label specific cells. Given the analogy between enteric glia and astrocytes, transgenic animals in which reporter proteins, such as green fluorescent protein (GFP) derivatives, have been placed under the direct control of *GFAP* or *S100*β regulatory elements to study astrocytic function can also be used to visualize enteric glial cells in live imaging experiments. Furthermore, the conditional expression of fluorescent reporters by Cre-Lox recombination technology enables identification of enteric glia as illustrated by Joseph et al. (2011), who combined *GFAP-Cre* (Zhuo et al., 2001) and *GFAP-CreER^T2^* (Hirrlinger et al., 2006) mice with *Rosa26ReYFP* reporter mice (Srinivas et al., 2001) for lineage tracing purposes. Time-dependent induction of Cre in the *Sox10-iCreER^T2^* transgenic mouse line generated by Laranjeira et al., not only allows fate mapping of multilineage ENS precursors and labeling of enteric neurons (Sasselli et al., 2013), but also elegantly enables marking enteric glial cells only (Laranjeira et al., 2011). A big advantage of such a genetic system is the fact that individual cells can be labeled, thus allowing appreciation of the cellular morphology as opposed to immunostaining of adjoining cells. These transgenic mouse lines can aid in the examination of enteric neuron-glia interactions in several ways. A non-exhaustive overview of mouse lines that could be used to visualize enteric glial cells is listed in Table 1.

**Table 1:** **Non-exhaustive list of mouse lines that can be used to visualize enteric glial cells**.

**Enteric glia**	**Mouse line**	**References**
**promoter**		
*S100β*	*S100β-GFP*	Vives et al., 2003; Zuo et al., 2004
*GFAP*	*GFAP-Cre*	Zhuo et al., 2001
	*GFAP-CreER^T2^*	Ganat et al., 2006; Hirrlinger et al., 2006
	*GFAP-GFP*	Zhuo et al., 1997; Kuzmanovic et al., 2003
	*GFAP-tTA*	Wang et al., 2004
	*GFAP-DsRed*	Noraberg et al., 2007
*Sox10*	*Sox10-iCreER^T2^*	Laranjeira et al., 2011; Simon et al., 2012

Tissue of each of these mouse lines (or in case of Cre and tTa lines: tissue from the correct offspring obtained from crosses with reporter lines) can be used either as a source of primary cell cultures, or to directly visualize enteric glia in whole mount preparations for live imaging, or finally, to combine with immunohistochemistry after fixation.

Fluorescent reporter lines are favorable over *post-hoc* immunohistochemistry for the identification and localization of glia in live imaging experiments since imaging can be performed directly in the cells of interest. It is for these reasons that *S100β-eGFP* mice have (Vives et al., 2003) been used in Ca^2+^ and nitric oxide (NO) imaging studies (Gulbransen and Sharkey, 2009; Lavoie et al., 2011; Maceachern et al., 2011). Also, ENS cultures have been generated from *Sox10-iCreER^T2^:R26R^FP635^* mice to characterize the Ca^2+^ responses of newborn neurons and genuine enteric glial cells upon a number of stimuli (Laranjeira et al., 2011). Here, the conditional expression of the red fluorescent protein FP635 (Shcherbo et al., 2009) was used to identify neurons that were derived from cultured enteric glial cells.

**Figure 5:**
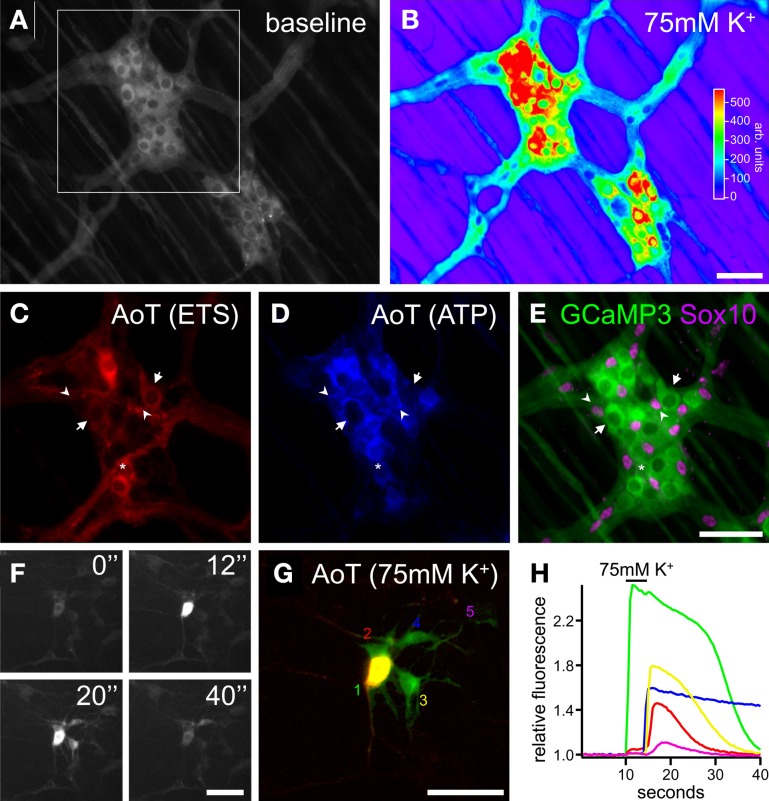
**Optogenetic Ca^2+^ imaging of enteric neuron-glia crosstalk. (A)** Gray scale image of myenteric plexus ganglia dissected from a *Wnt1-Cre;R26R-GCaMP3* mouse colon displaying baseline GCaMP3 fluorescence. **(B)** False-colored image of fluorescence response to 75 mM K^+^ depolarization (5 s) of the same ganglia as shown in **(A)**. **(C,D)** Activity over time (AoT) images of the myenteric ganglion in the frame indicated in **(A)** in which only pixels responding to electrical stimulation (ETS, 2 s, 20 Hz) of an interganglionic connective (**C**, red, see also Supplementary movie 2) or local ATP (10μM, 20 s) stimulation (**D**, blue, see also Supplementary movie 3) are shown. **(E)** GCaMP3 fluorescence image of the same ganglion as in **(C)** and **(D)** immunostained for Sox10 (magenta). Arrows indicate enteric neurons displaying a Ca^2+^ transient upon electrical stimulation only. Arrowheads point to enteric glial cells responding to ATP stimulation. Asterisk indicates an enteric neuron that responds to both electrical and purinergic stimulation. **(F)** Gray scale images of a patch of cultured myenteric neurons and glia established from a *Wnt1-Cre;R26R-GCaMP3* animal, before (0″), during (12″), and after (20″, 40″) stimulation with 75 mM K^+^ (5 s). **(G)** AoT image of the cells shown in **(F)** responding immediately (red) or with a delay (green) to 75 mM K^+^. See also Supplementary movie 4. **(H)** Recordings of the GCaMP3 responses to 75 mM K^+^ of one neuron and four surrounding enteric glial cells [color-coded numbers in **(G)**]. Neurons typically show an immediate Ca^2+^ transient to 75 mM K^+^ while enteric glial cells only respond with a delay, thus indicating neuron-to-glia communication. The slow downstroke of glial cell 4 (blue) could potentially be a sign of perturbed Ca^2+^ homeostasis or a general decline in cellular health. Note that *post-hoc* examination of cellular identity is redundant because of the genetically-imposed reporter expression. Scale bars: 50μm.

Another application of transgenic methods lies in the recent development of several optogenetic tools, an opportunity that has yet to be exploited in ENS research. The core instruments of these novel techniques are genetically-encoded optical indicators (Knopfel, 2012) and actuators (Fenno et al., 2011) that enable interrogation and manipulation of cell-to-cell interactions with cellular to subcellular resolution (Miesenbock, 2009). Among the optogenetic reporter molecules, genetically-encoded Ca^2+^ indicators (GECIs), such as GCaMPs, allow imaging of Ca^2+^ signaling in genetically defined cell populations, thus providing a powerful means to study neuron-glia interactions in the ENS. Recently, a reporter mouse was developed that expresses GCaMP3 (Tian et al., 2009) in a Cre-dependent manner in the *Rosa26* locus (Zariwala et al., 2012). By using the *Wnt1-Cre* transgene (Danielian et al., 1998) to conditionally express GCaMP3 in neural crest derivatives, we found that this system can also be used to perform Ca^2+^ imaging in enteric neurons and glia (Figure 5). Because tissue loading steps are omitted, tissue viability can be increased and background signals (e.g., from the underlying smooth muscle layers) reduced. However, to fully employ the advantages of these genetically-encoded indicators, they should ideally be expressed in enteric neurons or glial cells specifically. This will help to overcome the earlier illustrated problems caused by the close proximity between enteric neurons and glia. In addition, depending on the specifics of the transgenic method used, they can potentially enable monitoring of events in cellular subtypes. GECIs that tether to specific membrane proteins can be used to examine activity in thin glial processes and endfeet (Shigetomi et al., 2012, 2013). These are the cellular compartments that most likely interact with varicose fibers but are difficult to study using bulk loading dyes or normal cytosolic GECIs. This would yield important information about the signaling events in potential glial release sites and microdomains near the membrane.

## Imaging neuron-glia crosstalk in the human enteric nervous system

Because most investigations have been carried out using *in vitro* and *ex vivo* animal models, and given the difficulty to obtain healthy human gut tissues for experimental purposes, our current knowledge about enteric neuron–glia interactions in the human gut is rather poor. The limited information about human enteric glia function originates from *in vitro* studies using enteric glia isolated from surgical resection specimens. These studies indicated that human enteric glia actively participate in inflammatory responses (Cirillo et al., 2011) and host-bacteria crosstalk (Turco et al., 2013). However, even though these glial cells were obtained from ‘macroscopically normal' tissues, there is still the possibility that measurements were influenced by the fact that the resection specimens were collected from patients suffering from a variety of severe diseases.

For these reasons we have recently developed a method to culture human enteric glial cells isolated from routine intestinal biopsies (Boesmans et al., 2013). After careful removal of the mucosa, the submucosal plexus is enzymatically digested following previously described procedures (Cirillo et al., 2011), ganglia are selected and cells cultured on glass coverslips to perform live imaging studies. By implementing this technique we found that similar to rat enteric glia, also human enteric glial cells can be activated by neurotransmitters known to elicit fast excitatory responses in the ENS (Boesmans et al., 2013).

**Figure 6:**
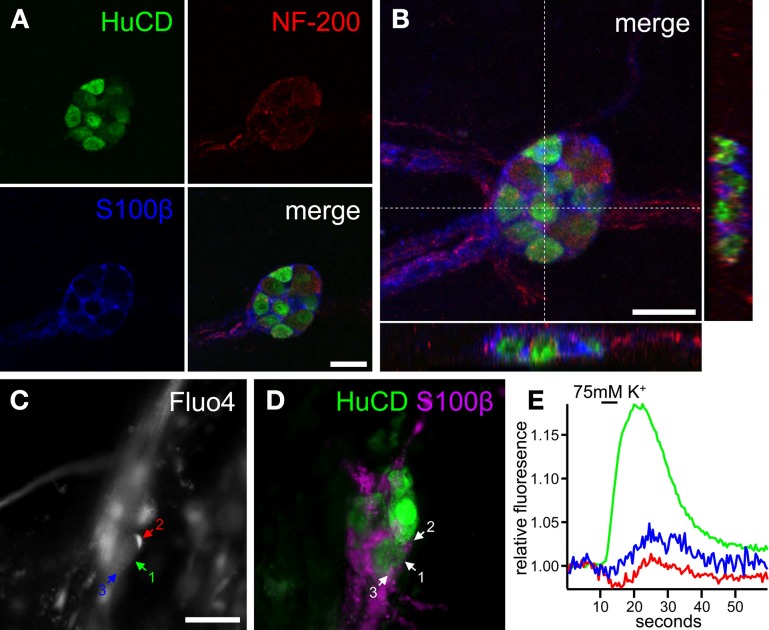
**Interactions between enteric neurons and glia in the human enteric nervous system. (A)** Individual confocal images of a human submucosal ganglion obtained from a duodenal biopsy in which enteric neurons are labeled with antibodies for HuCD (green) and NF-200 (red) and enteric glia are immunostained for S100β (blue). **(B)** Maximum projection of the same ganglion as in **(A)** with orthogonal X (bottom) and Y (right) views. Note how enteric neurons and glial cells are closely packed together within a dense ganglionic capsule. **(C)** Gray scale fluorescence image of a human submucosal ganglion loaded with the Ca^2+^ indicator Fluo4. **(D)** Image of the same ganglion as in **(C)**, immunostained for HuCD (green) and S100β (magenta). **(E)** Ca^2+^ responses of a neuronal cell body and two glial cell processes [color-coded numbers in **(C)** and numbers in **(D)**] upon 75 mM K^+^ depolarization. Scale bars: 25μm **(A,B)**, 50μm **(C)**.

Of course, in order to fully understand neuron-glia interactions in the human gut, also these should ideally be studied in intact *ex vivo* preparations. Although the interplay between enteric neurons and glia was not specifically envisaged, Mueller and colleagues were able to record enteric glial cell activity in resection specimens obtained from patients undergoing surgery (Mueller et al., 2011). With the recently developed optical imaging approach (Cirillo et al., 2013), we have shown that it is also feasible to record neuronal activity by means of Ca^2+^ imaging in submucosal ganglia dissected from duodenal biopsies obtained from healthy volunteers. This technique also allows exploring human enteric glia function (Figure 6). Again, analysis is not straightforward, even in comparison to the ENS of animal models, as the cells in the human enteric ganglia are organized in a far more three dimensional manner than in small animals. Nerve bundles and glial projections together form a complex structure (the ganglionic capsule) that surrounds neurons and glial cells (Figures 6A,B). Moreover, the presence of fasciculated bundles interconnecting adjacent ganglia adds to the intricacy of optical recordings from such ganglia. This makes correct interpretation of glial activation and discrimination between neuronal and glial signaling difficult, but not impossible. By analogy with the animal tissue experiments, it is still feasible, with careful attention to focus, movement, and analysis issues, to distinguish between signals originating from glia and neurons (Figures 6C–E). Here again, glial signals are delayed with respect to the responses observed in neuronal compartments.

## Conclusions and perspectives

Despite significant progress in understanding enteric glia function, the exact signaling mechanisms and possible “gliotransmitters𠄍 that act in a physiological context such as during the different gastrointestinal motility patterns remain elusive. Nonetheless, several studies indicate that enteric glial cells are active partners in enteric neurotransmission. In particular, the aforementioned live imaging studies have provided invaluable information about how enteric glial cells can detect neuronal activity (Gomes et al., 2009; Gulbransen and Sharkey, 2009; Gulbransen et al., 2010, 2012; Broadhead et al., 2012). As illustrated, the intimate association between enteric glia and neurons warrants careful experimental and analytic considerations. Enhancing optical resolution by using confocal recordings would obviously help toward avoiding false interpretation of the signal's origin especially when two structures truly overlap. However, this should be combined with deconvolution methods as resolution along the optical axis is never as high as in the XY plane. Confocal recordings can definitely improve the spatial resolution, but as we have shown, also timing is important to tell different responses apart, an advantage that is lost in classical point scanning confocals due to limitations in scan speed. Therefore, spinning disk confocal recordings may offer an intermediate solution. The toolbox of optical, off-line analysis and genetic approaches that we present here, can aid in disentangling the complex interplay between neurons and glia, thereby producing experimental access to bridge differing conclusions.

## Author contributions

Concept and design, interpretation of data, statistical analysis, drafting and editing of the manuscript were done by Werend Boesmans and Pieter Vanden Berghe; Werend Boesmans, Michiel A. Martens, Nathalie Weltens, and Marlene M. Hao performed experiments, image analysis and contributed to paper writing; Carla Cirillo performed and analyzed experiments on human enteric glia and was involved in paper writing; Jan Tack was responsible for mucosal biopsy collection; Pieter Vanden Berghe and Michiel A. Martens wrote analysis software; and Jan Tack and Pieter Vanden Berghe obtained funding.

## Conflict of interest statement

The authors declare that the research was conducted in the absence of any commercial or financial relationships that could be construed as a potential conflict of interest.
